# Gene Therapy for Non-Hereditary Retinal Disease: Age-Related Macular Degeneration, Diabetic Retinopathy, and Beyond

**DOI:** 10.3390/genes15060720

**Published:** 2024-06-01

**Authors:** Lucas W. Rowe, Thomas A. Ciulla

**Affiliations:** 1 Department of Ophthalmology, Indiana University School of Medicine, Indianapolis, IN 46202, USA; lwrowe@iu.edu; 2 Retina Service, Midwest Eye Institute, Indianapolis, IN 46290, USA

**Keywords:** gene therapy, anti-vascular endothelial growth factor (anti-VEGF), neovascular age-related macular degeneration (nAMD), diabetic macular edema (DME), diabetic retinopathy (DR)

## Abstract

Gene therapy holds promise as a transformative approach in the treatment landscape of age-related macular degeneration (AMD), diabetic retinopathy (DR), and diabetic macular edema (DME), aiming to address the challenges of frequent intravitreal anti-vascular endothelial growth factor (VEGF) injections. This manuscript reviews ongoing gene therapy clinical trials for these disorders, including ABBV-RGX-314, ixoberogene soroparvovec (ixo-vec), and 4D-150. ABBV-RGX-314 utilizes an adeno-associated virus (AAV) vector to deliver a transgene encoding a ranibizumab-like anti-VEGF antibody fragment, demonstrating promising results in Phase 1/2a and ongoing Phase 2b/3 trials. Ixo-vec employs an AAV2.7m8 capsid for intravitreal delivery of a transgene expressing aflibercept, showing encouraging outcomes in Phase 1 and ongoing Phase 2 trials. 4D-150 utilizes an evolved vector to express both aflibercept and a VEGF-C inhibitory RNAi, exhibiting positive interim results in Phase 1/2 studies. Other therapies reviewed include EXG102-031, FT-003, KH631, OLX10212, JNJ-1887, 4D-175, and OCU410. These therapies offer potential advantages of reduced treatment frequency and enhanced safety profiles, representing a paradigm shift in management towards durable and efficacious cellular-based biofactories. These advancements in gene therapy hold promise for improving outcomes in AMD and addressing the complex challenges of DME and DR, providing new avenues for the treatment of diabetic eye diseases.

## 1. Background

**Figure 1:**
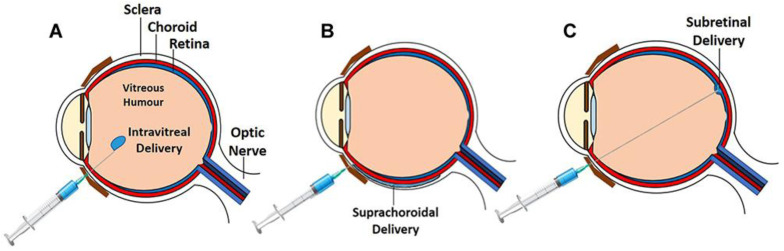
Routes of ocular gene therapy administration: subretinal delivery (**A**); intravitreal delivery (**B**); and suprachoroidal delivery (**C**). From Ghoraba HH, Akhavanrezayat A, Karaca I, et al. *Clin. Ophthalmol*. 2022; 16:1753–71 [6]. Licensed for reuse under the Creative Commons Attribution—Non-Commercial (unported, v3.0) license.

Retina has been at the forefront of gene therapy in medicine, particularly since the U.S. Food and Drug Administration (FDA) clearance of voretigene neparvovec-rzyl (Luxturna) for confirmed biallelic RPE65-mediated inherited retinal disease in December 2017. Luxturna represented the first FDA-approved gene therapy for inherited disease. The landscape of gene therapy in retina continues to swiftly advance and has garnered much attention for its potential in non-hereditary retinal disease. When a therapeutic gene is introduced and integrates into patient cells, it can continuously produce the desired protein, such as endogenous anti-vascular endothelial growth factor (VEGF). This approach promises to reduce the need for frequent intravitreal injections by providing sustained and long-lasting therapeutic effects. Subretinal injection is the standard method for ocular gene therapy administration, but alternative routes like suprachoroidal and intravitreal delivery are being explored to avoid complications associated with pars plana vitrectomy procedures. These complications can include macular holes, potential photoreceptor damage due to bleb creation, retinotomy with hemorrhage and fibrosis, retinal tears and detachment, and cataracts (Figure 1) [1]. Suprachoroidal delivery offers targeted, compartmentalized delivery to the chorioretina while reducing exposure to the vitreous and anterior segment, potentially decreasing immune and inflammatory responses [2,3,4,5]. These gene therapy delivery methods are under investigation for treating various non-hereditary retinal diseases, particularly age-related macular degeneration (AMD), diabetic macular edema (DME), and diabetic retinopathy (DR) (Table 1).

## 2. Age-Related Macular Degeneration

AMD represents the leading cause of legal blindness in individuals over 50 years of age in developed nations [7,8], including a global prevalence of up to 8.7%, as demonstrated in several population-based studies. The prevalence of AMD is expected to increase significantly with the aging global population to almost 300 million people by 2040 [9]. AMD is a complex condition marked by the formation and buildup of extracellular deposits known as drusen in the subretinal space. This leads to a gradual deterioration of the retinal pigment epithelium (RPE), photoreceptors, and nearby tissues, ultimately resulting in the loss of central vision. AMD is classified into two forms: non-exudative (non-neovascular, dry) AMD and exudative or neovascular AMD (nAMD).

### 2.1. Wet Age-Related Macular Degeneration

**Figure 2:**
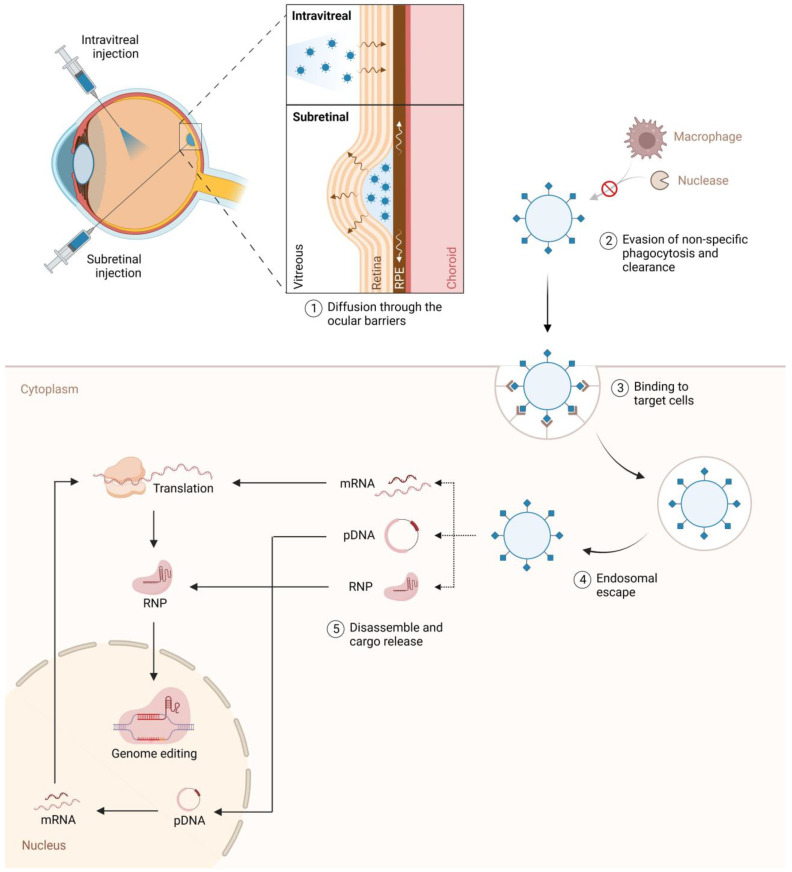
Overview of adenovirus-mediated delivery of recombinant genetic material to host cells for endogenous expression of a desired protein. From Carvalho C, Lemos L, Antas P, Seabra MC. *Front. Ophthalmol*. 2023; 3:1270561 [29]. Licensed for reuse under the Creative Commons Attribution License (CC BY) license.

nAMD is characterized by choroidal neovascularization (CNV) and accounts for approximately 20% of AMD and nearly 90% of severe vision loss from AMD [10,11]. Over the past decade, intravitreal anti-vascular endothelial growth factor (VEGF) injections have become the standard of care for the management of nAMD [12]. Several large, prospective, randomized, controlled multicenter studies have established the visual benefits of intravitreal anti-VEGF drugs for nAMD [13,14,15,16,17,18,19,20,21,22,23]; however, real-world data indicate that patients often experience fewer injections and less improvement in best-corrected visual acuity (BCVA) compared to those in controlled trials [24,25,26]. Patients on continuous fixed-interval dosing with over ten injections annually tend to maintain better long-term BCVA [27], while those who receive fewer than six injections per year demonstrate less visual improvement [28]. Frequent clinic visits and injections represent a significant treatment burden for patients, caregivers, and providers. Therefore, there is a pressing need for the development of longer-acting therapies and innovative technologies for treating nAMD. Gene therapy has the capacity to continually generate the desired protein, such as endogenous anti-VEGF, and thus offers the prospect of alleviating the treatment burden of frequent intravitreal injections through sustained and enduring therapeutic effects (Figure 2).

ABBV-RGX-314 is a gene therapy being developed by REGENXBIO (Rockville, MD, USA) and AbbVie (North Chicago, IL, USA) that utilizes an adeno-associated virus (AAV) 8 vector that delivers a transgene encoding a ranibizumab-like anti-VEGF monoclonal antibody fragment to the retina. The therapy is currently being investigated as a single subretinal or suprachoroidal injection to produce enduring cellular expression of the anti-VEGF protein. The results of a Phase 1/2a, open-label, multiple-cohort, dose-escalation study were recently published and reported positive safety and efficacy results with a single administration of subretinal ABBV-RGX-314 in nAMD. ABBV-RGX-314 was well tolerated at all five doses and demonstrated stability of BCVA and central retinal thickness (CRT) up to two years [30,31]. The results of this study support two ongoing Phase 2b/3 trials, ATMOSPHERE and ASCENT, investigating subretinal ABBV-RGX-314 in nAMD [32,33]. The ATMOSPHERE (Phase 2b/3) and ASCENT (Phase 3) trials are randomized, partially masked, controlled studies evaluating the mean change in BCVA from baseline to 54 weeks of two dose levels of subretinal ABBV-RGX-314 against monthly intravitreal ranibizumab and bimonthly intravitreal aflibercept, respectively. REGENXBIO expects the trials to support global regulatory submission in late 2025 and the first half of 2026 [34].

**Figure 3:**
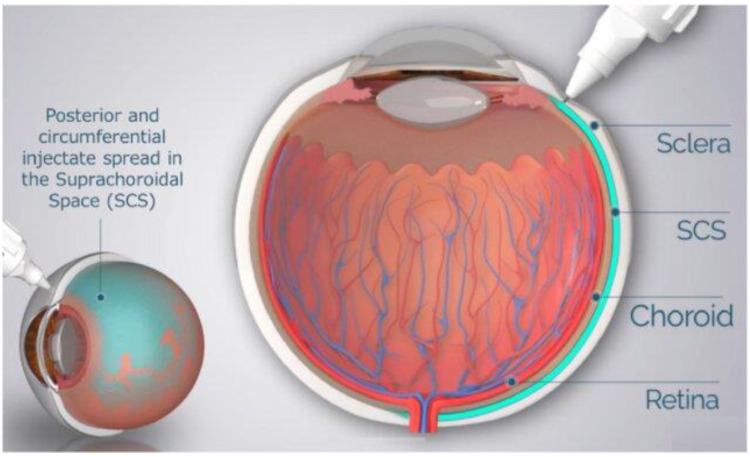
Schematic of microneedle injection into the suprachoroidal space (SCS). From Wan C-R, Muya L, Kansara V, Ciulla TA. *Pharmaceutics*. 2021; 13:288 [2]. Licensed for reuse under the Creative Commons Attribution (CC BY) license.

REGENXBIO is also evaluating the suprachoroidal delivery of ABBV-RGX-314 in individuals with nAMD. The AAVIATE Phase 2 trial is a multicenter, open-label, randomized, active-controlled, dose-escalation study investigating the efficacy, safety, and tolerability of suprachoroidal ABBV-RGX-314 using the Clearside Suprachoroidal Space (SCS) Microinjector in comparison to monthly intravitreal ranibizumab (Figure 3). In the trial, ABBV-RGX-314 is administered at three dose levels: 2.5 × 10^11^ (Cohort 1), 5 × 10^11^ (Cohorts 2 and 3), and 1 × 10^12^ (Cohorts 4–6) genomic copies per eye. The primary efficacy endpoint is the mean change in BCVA from baseline to week 40, with secondary endpoints including the mean change in CRT and number of anti-VEGF injections following administration. Interim data released by REGENXBIO revealed that over 50 patients in Cohorts 4–6 achieved an 80% reduction in the annualized injection rate (i.e., mean number of anti-VEGF injections per year) and a 50% injection-free rate through 6 months following a single suprachoroidal injection of ABBV-RGX-314 [35]. Moreover, the treatment was well tolerated in over 100 patients across the three dose levels with no drug-related serious adverse events. Mild intraocular inflammation (IOI) occurred at similar rates in the first and second dose levels. Cohorts 4 and 5 at the third dose level had reports of mild to moderate inflammation, all of which resolved with topical corticosteroids. Cohort 6 included patients at the third dose level who received a short course of prophylactic topical steroids and there were no cases of IOI [35]. These early results support the promising potential of ABBV-RGX-314 as a single-session, in-office therapy that may offer long-term efficacy and safety in nAMD.

Ixoberogene soroparvovec (ixo-vec, formerly ADVM-022) (Adverum Biotechnologies Inc., Redwood City, CA, USA) is an intravitreal gene therapy utilizing the AAV2.7m8 capsid to efficiently deliver and express aflibercept [36,37]. OPTIC, a two-year, open-label, multicenter Phase 1 trial, aimed to assess ixo-vec’s safety and tolerability in non-naïve nAMD patients [38]. Participants were distributed across four cohorts differing by ixo-vec dosage (2 × 10^11^ or 6 × 10^11^ vector genomes per eye, vg/eye) and either oral prednisone or topical difluprednate as prophylactic steroids. The majority of ocular treatment-emergent adverse events (TEAEs) were mild (84%) to moderate (16%) and dose-dependent, with anterior chamber cell and vitreous cell being the most commonly reported [39]. Among the five reported serious TEAEs, two were possibly related to ixo-vec, including asymmetric dry AMD progression and recurrent uveitis. Importantly, the high-dose group and low-dose group experienced reductions in annualized anti-VEGF injections of 98% and 80% and injection-free rates of 80% and 53%, respectively [39]. Both dosage groups maintained BCVA and CRT measurements throughout the study. The OPTIC trial’s findings supported further evaluation of the 2 × 10^11^ vg/eye dose in nAMD due to its superior safety profile and comparable efficacy to the higher dose.

The ongoing Phase 2 LUNA trial is investigating the safety and efficacy of ixo-vec at the 2 × 10^11^ vg/eye dose and a lower dose of 6 × 10^10^ vg/eye, in combination with enhanced corticosteroid prophylaxis. Adverum recently disclosed promising preliminary findings from the LUNA study, with both doses maintaining visual and anatomic outcomes. At 26 weeks, the 2 × 10^11^ and 6 × 10^10^ vg/eye doses achieved annualized reduction in anti-VEGF injection rates of 94% and 90% and injection-free rates of 85% and 68%, respectively [40]. Both doses demonstrated sustained functional and anatomical improvements through 26 weeks, in addition to apparent improved inflammatory profiles with corticosteroid prophylaxis compared to those from the OPTIC study. These early results support the potential of a single in-office intravitreal injection of ixo-vec to revolutionize nAMD treatment by offering durable and effective cellular-based biofactories, reducing the need for frequent anti-VEGF injections.

Furthermore, 4D-150 (4D Molecular Therapeutics, 4DMT, Emeryville, CA, USA) is a single-use, low-dose intravitreal gene therapy employing the R100 vector to deliver a transgene cassette that expresses both aflibercept and a VEGF-C inhibitory RNAi to inhibit a total of four antiangiogenic factors: VEGF A, B, and C and placental growth factor (PlGF) [41]. PRISM is a prospective, multicenter, Phase 1/2 dose-escalation and randomized, controlled, masked expansion trial investigating the safety and tolerability of 4D-150 in nAMD [42]. Phase 1 results demonstrated all three dose cohorts of 3 × 10^10^, 1 × 10^10^, and 6 × 10^9^ vg/eye of 4D-150 were safe and well tolerated, with the 3 × 10^10^ vg/eye cohort experiencing a 96.7% overall reduction in the mean annualized anti-VEGF injection rate and four of five eyes becoming supplemental-aflibercept-free [43]. Interim Phase 2 results met all key endpoints through 24 weeks, and the 3 × 10^10^ vg/eye cohort demonstrated equivalent and stable BCVA outcomes and improved retinal anatomical control with reduced central subfield thickness variability compared to the bimonthly aflibercept arm, in addition to an 89% overall reduction in the annualized anti-VEGF injection rate and a 63% injection-free rate. Notably, a favorable safety profile was observed with no significant or recurrent IOI with a 20-week prophylactic topical corticosteroid taper. Following the positive initial results from the PRISM trial, 4DMT received RMAT and Priority Medicines (PRIME) designations from the FDA and European Medicines Agency (EMA), respectively, with the goal to increase collaboration on regulatory approval planning and expediting drug development [41].

EXG102-031 (Exegenesis Bio, Lower Gwynedd Township, PA, USA) is a subretinal injection of recombinant AAV (rAAV)-based gene therapy that expresses an angiopoietin (Ang) domain and VEGF receptor (ABD-VEGFR) fusion protein which binds and neutralizes all known subtypes of VEGF and Ang-2 [44]. EXG102-031 is currently undergoing an open-label, dose-escalation Phase 1/2a study designed to evaluate its safety and efficacy in nAMD [45].

FT-003 (Frontera Therapeutics, Bedford, MA, USA) is a subretinal injection of an AAV gene expression system using a novel manufacturing platform to provide durable expression of therapeutic levels of intraocular protein. FT-003 is being investigated in an open-label, single-center, dose-escalation trial to evaluate its safety, tolerability, and preliminary efficacy in nAMD subjects [46,47].

KH631 (Chengdu Origen Biotechnology, Sichuan, China; Vanotech, Rockaway, NJ, USA) is a subretinal rAAV8 vector injection engineered to produce a human VEGF receptor fusion protein consisting of domain 2 of VEGFR1, domain 3 and domain 4 of VEGFR2, and the Fc domain of human immunoglobulin (Ig)G1 with binding affinity to VEGF-A, VEGF-B, and PlGF. In preclinical studies using non-human primates, subretinal delivery of KH631 at a low concentration of 3 × 10^8^ vg/eye demonstrated significant retention of the therapeutic protein within the retina, halting the development and advancement of grade IV CNV lesions, which are regarded as a proxy for active nAMD in humans [48]. Moreover, sustained expression of the therapeutic gene was observed for over 96 weeks. The VAN-2201 clinical trial is an ongoing, multicenter, open-label, dose-escalating clinical trial to assess the safety and tolerability of KH631 in five dose cohorts in subjects with nAMD [49].

OLX10212 (OliX Pharmaceuticals, Suwon, Republic of Korea) is a cell-penetrating asymmetric small interfering RNA (cp-asiRNA) administered via a single intravitreal injection that targets inflammatory pathways upstream of VEGF. A current, multicenter, single-dose, dose-escalating Phase 1 study is evaluating the safety and tolerability of OLX10212 at dose levels between 100 μg/eye/50 μL and 950 μg/eye/50 μL [50]. Olix Pharmaceuticals recently announced positive safety data and preliminary efficacy results from this study, including no signs of inflammation, changes in intraocular homeostasis, or systemic effects in all subjects. The study has also identified dose levels suitable for evaluations of efficacy testing in future clinical trials [50].

JNJ-1887 (JNJ-81201887, HMR-59, Janssen Pharmaceuticals, Beerse, Belgium) is an intravitreal anti-complement gene-based therapy that utilizes an AAV2 to endogenously increase the expression of a soluble form of CD59 (sCD59). CD59 inhibits the formation of the membrane attack complex (MAC), a process implicated in AMD pathogenesis, showing correlation with disease severity and damage to retinal pigment epithelium (RPE). JNJ-1887 is under development for the treatment of nAMD and geographic atrophy (GA) secondary to dry AMD. Two Phase I trials, 1001 and 1002, demonstrated the safety of JNJ-1887 in patients with GA and nAMD, respectively [51]. Trial 1002 was an open-label, multicenter study over 12 months that enrolled 25 subjects with treatment-naïve nAMD. Subjects received an initial anti-VEGF injection followed by a single intravitreal injection of JNJ-1887 at doses of 3.56 × 10^11^ or 1.071 × 10^12^ vg/eye, with a protocol-defined oral corticosteroid prophylaxis regimen. Four cases of ocular inflammation occurred, all of which were mild or moderate in severity and resolved following a short course of oral steroids and topical steroids of varied duration. Preliminary results revealed that 18.2% of subjects treated with the lower dose did not require re-treatment in the first 6 months [52]. These early findings suggest that JNJ-1887 may offer a therapeutic advantage over other treatments for patients experiencing both CNV and GA concurrently in one eye, a situation documented in various clinical investigations [53]. Anti-VEGF therapy has also been associated with the development of GA, complicating the management of individuals with both pathways involved.

The promise of gene therapy in nAMD lies in its potential to ease treatment burdens by providing sustained therapeutic effects through the endogenous production of anti-VEGF within the retina. While challenges persist in the development of these therapies, advancements in administration routes, safety, and efficacy are notable. The potential reduction in treatment frequency could greatly benefit patients, improving quality of life, preserving vision, and lessening the socioeconomic impact of vision loss. Viral vector gene therapy stands out as a promising approach, underscoring the significant progress in reshaping nAMD treatment strategies.

### 2.2. Dry Age-Related Macular Degeneration

**Figure 4:**
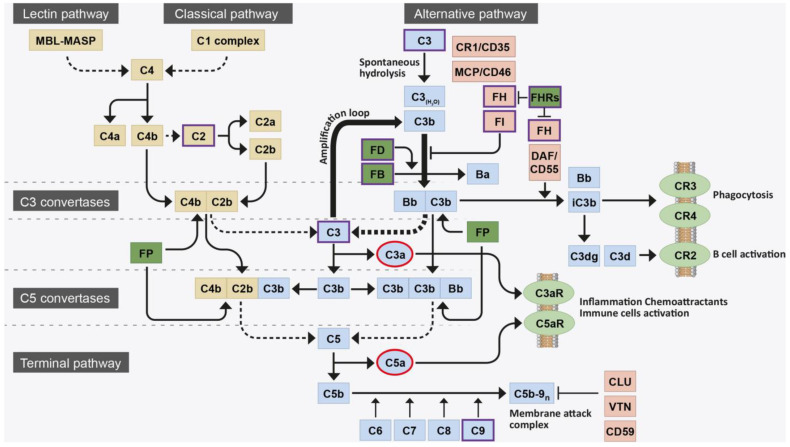
Overview of the complement system and its activation pathway involved in the pathogenesis of dry age-related macular degeneration. From Armento A, Ueffing M, Clark SJ. *Cell Mol Life Sci*. 2021; 78(10):4487-4505 [59]. Licensed for reuse under the Creative Commons Attribution-ShareAlike 4.0 International license.

GA represents an advanced manifestation of dry AMD, marked by the degeneration of photoreceptors, retinal pigment epithelium, and choriocapillaris. This condition leads to significant, irreversible visual impairment in individuals aged 60 years or older, with approximately 5 million people affected by GA globally [9,54]. The burden of illness associated with geographic atrophy is considerable, greatly diminishing the quality of life for both patients and their caregivers [55,56]. The landscape of treating GA associated with advanced dry AMD has swiftly evolved due to the recent approval of two intravitreal complement inhibitors: pegcetacoplan (Syfovre, Apellis Pharmaceuticals, Waltham, MA, USA) and avacincaptad pegol (Izveray, Astellas Pharma, Chuo City, Tokyo, Japan) [57,58]. These medications act by inhibiting the complement cascade, which is implicated in the onset of retinal pigment epithelium cell death resulting in GA (Figure 4). They are limited, however, by the necessity of indefinite monthly or bimonthly administration and an increased risk of conversion to nAMD. Gene therapy has garnered great interest for its potential as a longer-lasting therapeutic option for GA.

As previously presented, JNJ-1887 is an intravitreal anti-complement gene-based therapy that utilizes an AAV2 vector to endogenously increase the expression of sCD59. In addition to nAMD, JNJ-1887 is under development for the treatment of GA secondary to dry AMD. Trial 1001 was an open-label, single-center study over 24 months that enrolled 17 subjects with GA in three dose groups: low (3.56 × 10^10^ vg/eye; n = 3), intermediate (1.07 × 10^11^ vg/eye; n = 3), and high (3.56 × 10^11^ vg/eye; n = 11) [51]. Mild IOI occurred in five patients; these cases were managed through monitoring until resolution or topical corticosteroid treatment. Although not powered to evaluate efficacy, the trial found that most subjects in the high-dose group exhibited a rate of GA progression slower than historical controls, while two patients experiencing inflammation displayed increased growth [52]. Furthermore, there were no cases of conversion of nAMD.

Janssen Pharmaceuticals is currently enrolling patients with GA secondary to dry AMD in its Phase IIb PARASOL trial [60]. The company plans to enroll 300 participants in three cohorts: a single low-dose intravitreal injection of JNJ-1887 with oral prednisone and single periocular triamcinolone injection prophylaxis, a single high-dose intravitreal injection of JNJ-1887 with oral prednisone and single periocular triamcinolone injection prophylaxis, and a placebo group. The primary outcome to be measured is the change from baseline in the square root of GA lesion area through 18 months. JNJ-1887 has been granted Fast Track designation by the FDA and Advanced Therapy Medicinal Product (ATMP) designation by the European Medicines Agency (EMA) [61].

In addition, 4D-175 (formerly sCFH, 4D Molecular Therapeutics, Emeryville, CA, USA) is a candidate therapy for GA utilizing 4DMT’s proprietary retinotropic R100 vector to engineer the transgene encoding short-form human complement factor H (sCFH). sCFH is a shortened and optimized form of complement Factor H (CFH), a master inhibitor and regulator of the inflammatory complement system. CFH gene mutations have been identified as significant genetic risk factors for AMD, including GA. Approximately 75% of GA patients carry high-risk CFH variants, which reduce complement inhibitory function and result in hyperactive complement pathway activity. Furthermore, 4DMT has announced a press release revealing that an Investigational New Drug (IND) filing is expected in Q2 2024 with Phase 1 initiation expected in H2 2024 [62]. 

OCU410 (AAV-hRORA, Ocugen, Malvern, PA, USA) employs an AAV delivery system to transport the RORA (RAR Related Orphan Receptor A) gene into the retina. RORA protein functions crucially in lipid metabolism, diminishing lipofuscin deposits and oxidative stress. Additionally, it exhibits anti-inflammatory properties and suppresses the complement system, as evidenced in both in in vitro and animal model studies [63,64]. The ArMaDa Phase 1/2 trial aims to evaluate the safety of unilateral subretinal administration of OCU410 in individuals with GA and will be conducted in two phases. Phase 1 involves a multicenter, open-label, dose-ranging study comprising three dose levels: low dose (2.5 × 10^10^ vg/mL), medium dose (5 × 10^10^ vg/mL), and high dose (1.5 × 10^11^ vg/mL). Phase 2 is a randomized, outcome-accessor-blinded, dose-expansion study that will randomly assign subjects in a 1:1:1 ratio to either one of two OCU410 treatment groups or to an untreated control group. Ocugen recently announced that subjects in the low-dose cohort have experienced no serious adverse events related to OCU410 to date and that dosing in the medium cohort has been completed [65,66].

## 3. Diabetic Retinopathy and Diabetic Macular Edema

Diabetic retinopathy (DR) and diabetic macular edema (DME) represent the primary causes of blindness in individuals with diabetes. DR is clinically categorized into two main types: non-proliferative DR (NPDR) and proliferative DR (PDR). NPDR, an early phase of DR, is marked by increased vascular permeability and capillary blockages, as evidenced by microaneurysms, hemorrhages, and hard lipid exudates on fundus examination. PDR, on the other hand, is an advanced stage characterized by the growth of abnormal vessels into the vitreous scaffold, often resulting in vitreous hemorrhage and sudden vision loss, particularly following vitreous traction. Ultimately, neovascularization can lead to fibrosis and contraction on the inner retinal surface, culminating in tractional retinal detachment. DME, another complication of DR, can manifest at any stage and involves macular thickening due to fluid accumulation in the sub- and intra-retinal spaces, leading to decreased visual acuity and central visual distortion (metamorphopsia) [67,68]. According to published data, it is estimated that around one million individuals in the United States have DME. DME is currently managed with intravitreal injections of anti-VEGF agents, typically administered every 4–12 weeks.

### 3.1. Diabetic Macular Edema

Many of the same gene therapy candidates for nAMD are being investigated in diabetic macular edema. For example, 4D-150 is a single-use, low-dose intravitreal gene therapy employing the R100 vector to deliver a transgene cassette that expresses both aflibercept and a VEGF-C inhibitory RNAi. The Phase 2 SPECTRA trial is a multicenter, randomized, active-controlled, double-masked, dose-ranging trial to evaluate the safety and efficacy of intravitreal 4D-150 in adults with DME [69,70]. The SPECTRA trial consists of two stages. The dose confirmation stage consists of randomization of subjects 1:1:1 to receive a single intravitreal injection of 5 × 10^9^ or 1 × 10^10^ vg/eye of 4D-150 (initial dose levels) or aflibercept control. The dose expansion stage includes the randomization of eligible participants 1:1:1 to receive a single intravitreal injection of 4D-150 at one of two dose levels based on the results from the dose confirmation stage or aflibercept control. Encouragingly, enrollment and dosing in the dose confirmation stage was announced as completed, with initial interim 24-week landmark data analysis expected in H2 2024 [71]. Preliminary results from the study are highly anticipated to better understand the therapy’s potential in DME.

FT-003 is a subretinal injection of an AAV gene expression system developed to ensure the long-lasting production of therapeutic levels of intraocular protein. Frontera is enrolling and dosing patients with three dose levels of FT-003 to investigate its safety, tolerability, and preliminary efficacy in DME subjects [72,73].

### 3.2. Diabetic Retinopathy

In addition to nAMD, ABBV-RGX-314 is presently under investigation for treating diabetic retinopathy without center-involving diabetic macular edema. The Phase 2 ALTITUDE trial is an open-label, controlled, dose-escalation trial evaluating the efficacy, safety, and tolerability of suprachoroidal ABBV-RGX-314 at two dose levels (2.5 × 10^11^ GC/eye and 5 × 10^11^ GC/eye) in three cohorts in patients with a DR diagnosis of moderately severe or severe NPDR or mild PDR. At 1 year, both doses of ABBV-RGX-314 were well tolerated, with no cases of chorioretinitis, vasculitis, occlusion, or hypotony. There were a few mild and transient cases of episcleritis and IOI; however, they occurred in the cohorts without prophylactic steroids and responded well to topical therapy [74]. Subjects in cohorts 4 and 5, who received topical prophylactic steroids, displayed no cases of IOI. Notably, 100% of subjects with baseline NPDR who received the higher dose level demonstrated stable to improved disease severity, with 70.8% achieving any improvement in diabetic retinopathy severity scale (DRSS) score compared to 25.0% in the control group. Furthermore, none of these patients demonstrated a 2+ step worsening of the DRSS score compared to 37.5% of control patients. Lastly, the higher dose reduced the risk of developing vision-threatening events by 89% in these patients [74].

## 4. Conclusions

In conclusion, the field of gene therapy for retinal diseases, particularly nAMD, is rapidly evolving, offering promising alternatives to the current standard of care. The ongoing clinical trials highlighted in this manuscript demonstrate the potential of gene therapies such as ABBV-RGX-314, ixo-vec, and 4D-150 to reduce treatment burden, improve patient outcomes, and revolutionize the management of nAMD. Furthermore, the extension of gene therapy research to DME and DR represents a significant step forward in addressing the complex challenges associated with diabetic eye diseases. These advancements not only offer hope for improved visual outcomes but also underscore the potential of gene therapy as a transformative approach in the treatment of retinal diseases.

However, it is essential to acknowledge the challenges and limitations that accompany the development and implementation of gene therapies. Factors such as safety concerns, optimal dosing regimens, long-term efficacy, and accessibility to these innovative treatments warrant further investigation and consideration. Additionally, the complexities of delivering gene therapies, including route of administration and potential immune responses, underscore the need for continued research and refinement in this field.

As gene therapy for retinal diseases progresses from clinical trials to real-world applications, collaboration between researchers, clinicians, industry partners, and regulatory agencies will be crucial in ensuring the safe and effective integration of these therapies into clinical practice. Furthermore, ongoing efforts to address the socioeconomic impact of vision impairment and increase accessibility to innovative treatments will be paramount in maximizing the potential benefits of gene therapy for patients with retinal diseases.

In summary, while challenges remain, the advancements in gene therapy presented in this manuscript offer a glimpse into a future where the treatment landscape for retinal diseases is transformed, providing hope for improved outcomes and quality of life for patients worldwide.

## Figures and Tables

**Table 1:** Summary of current gene therapies for non-hereditary retinal diseases in development.

Drug (Company)	Disease Indication	Delivery Technique	Mechanism of Action	Phase of Development	Treatment Effect	Safety Profile
ABBV-RGX-314 (REGENXBIO, AbbVie)	nAMD and DR	Subretinal (nAMD) and suprachoroidal (nAMD and DR)	AAV8 vector: transgene encoding a ranibizumab-like anti-VEGF monoclonal antibody fragment	nAMD: Subretinal: Phase 2b/3 ATMOSPHERE and Phase 3 ASCENT trials; suprachoroidal: Phase 2 AAVIATE trial DR: Phase 2 ALTITUDE trial	nAMD: Subretinal: stability of BCVA and CRT up to two years; suprachoroidal: 80% reduction in annualized injection rate and 50% injection-free rate through six months DR: higher dose level demonstrated stable to improved DRSS in in 100% of patients with baseline NPDR, higher dose reduced the risk of developing vision-threatening events by 89%	nAMD: Subretinal: well tolerated at all five doses; Suprachoroidal: mild to moderate IOI which resolved with topical steroids and no cases of IOI in cohort that received a short course of prophylactic topical steroids DR: few cases of mild and transient episcleritis and IOI which resolved with topical steroids and no cases of IOI in cohorts that received a short course of prophylactic topical steroids
Ixoberogene soroparvovec (Adverum Biotechnologies)	nAMD	Intravitreal	AAV2.7m8 capsid: transgene expressing aflibercept	Phase 2 LUNA trial	Sustained functional and anatomical improvements through 26 weeks; annualized reduction in anti-VEGF injection rates of 94% and injection-free rate of 85%	Improved inflammatory profiles with corticosteroid prophylaxis compared to those from the Phase 1 OPTIC study
4D-150 (4D Molecular Therapeutics)	nAMD and DME	Intravitreal	R100 vector: transgene cassette that expresses both aflibercept and a VEGF-C inhibitory RNAi	nAMD: Phase 2; DME: Phase 2 SPECTRA trial	nAMD: 89% overall reduction in annualized anti-VEGF injection rate and a 63% injection-free rate through 24 weeks; DME: pending preliminary results	All three dose cohorts were safe and well tolerated
EXG102-031 (Exegenesis Bio)	nAMD	Subretinal	rAAV: transgene expresses ABD-VEGFR fusion protein (binds and neutralizes all known subtypes of VEGF and Ang-2)	Phase 1/2a trial	Pending preliminary results	Pending preliminary results
FT-003 (Frontera Therapeutics)	nAMD and DME	Subretinal	AAV gene expression system using a novel manufacturing platform: expression of undisclosed intraocular protein	Phase 1 trial	Pending results	Pending results
KH631 (Chengdu Origen Biotechnology, Vanotech)	nAMD	Subretinal	rAAV8 vector: human VEGF receptor fusion protein with binding affinity to VEGF-A, VEGF-B, and PlGF	Phase 1 VAN-2201 trial	Pending results	Pending results
OLX10212 (OliX Pharmaceuticals)	nAMD	Intravitreal	cp-asiRNA: targets inflammatory pathways upstream of VEGF	Phase 1 trial	Preliminary BCVA improvement	No signs of inflammation or changes in intraocular homeostasis noted in all patients
JNJ-1887 (Janssen Pharmaceuticals)	nAMD and dry AMD	Intravitreal	AAV2 vector: expression of sCD59	nAMD: Phase 1 1002 trial; GA: Phase IIb PARASOL trial	nAMD: 18.2% of subjects treated with the lower dose did not require re-treatment in the first 6 months; GA: most subjects in the high-dose group exhibited a rate of GA progression slower than historical controls, while two patients experiencing inflammation displayed increased growth; no cases of conversion to nAMD	nAMD: mild or moderate cases of IOI which resolved following a short course of oral steroids and topical steroids of varied duration; GA: mild IOI which resolved through monitoring or topical steroids
4D-175 (4D Molecular Therapeutics)	Dry AMD	Intravitreal	R100 vector: transgene encoding sCFH	Phase 1 pending initiation	Pending results	Pending results
OCU410 (Ocugen)	Dry AMD	Subretinal	AAV vector: RORA gene delivery	Phase 1/2 ArMaDa trial	Pending results	No serious adverse events in the low-dose cohort

Abbreviations: AAV, adeno-associated virus; ABD, angiopoietin (Ang) domain; AMD, age-related macular degeneration; BCVA, best-corrected visual acuity; CRT, central retinal thickness; cp-asiRNA, cell-penetrating asymmetric small interfering RNA; DME, diabetic macular edema; DR, diabetic retinopathy; DRSS, diabetic retinopathy severity scale; GA, geographic atrophy; IOI, intraocular inflammation; nAMD, neovascular age-related macular degeneration; PlGF, placental growth factor; rAAV, recombinant adeno-associated virus; RORA, RAR Related Orphan Receptor A; sCFH, short-form human complement factor H; sCD59, soluble form of CD59; VEGF, vascular endothelial growth factor; VEGFR, vascular endothelial growth factor receptor.

## Data Availability

No new data were created or analyzed in this study. Data sharing is not applicable to this article.
